# Recent Advances in Lentiviral Vaccines for HIV-1 Infection

**DOI:** 10.3389/fimmu.2016.00243

**Published:** 2016-06-21

**Authors:** Thomas D. Norton, Elizabeth A. Miller

**Affiliations:** ^1^Department of Medicine, Division of Infectious Diseases, NYU School of Medicine, New York, NY, USA; ^2^Department of Medicine, Division of Infectious Diseases, Icahn School of Medicine at Mount Sinai, New York, NY, USA

**Keywords:** dendritic cells, lentiviral vectors, HIV-1, HIV-1 vaccines, SAMHD1, Vpx

## Abstract

The development of an effective HIV vaccine to prevent and/or cure HIV remains a global health priority. Given their central role in the initiation of adaptive immune responses, dendritic cell (DC)-based vaccines are being increasingly explored as immunotherapeutic strategies to enhance HIV-specific T cells in infected individuals and, thus, promote immune responses that may help facilitate a functional cure. HIV-1-based lentiviral (LV) vectors have inherent advantages as DC vaccine vectors due to their ability to transduce non-dividing cells and integrate into the target cell genomic DNA, allowing for expression of encoded antigens over the lifespan of the cell. Moreover, LV vectors may express additional immunostimulatory and immunoregulatory proteins that enhance DC function and direct antigen-specific T cells responses. Recent basic and clinical research efforts have broadened our understanding of LV vectors as DC-based vaccines. In this review, we provide an overview of the pre-clinical and clinical LV vector vaccine studies for treating HIV to date. We also discuss advances in LV vector designs that have enhanced DC transduction efficiency, target cell specificity, and immunogenicity, and address potential safety concerns regarding LV vector-based vaccines.

## Introduction

Although antiretroviral therapy has dramatically improved the outcome of HIV infection (1, 2), it is not curative. The failure to cure HIV is due to a reservoir of latently infected T cells formed in the acute phase of infection that re-establishes virus loads upon treatment interruption, thus necessitating lifelong therapy. The development of a vaccine to prevent or cure HIV is, therefore, a major public health priority to fight the global epidemic.

There have been extensive efforts to develop an effective HIV vaccine; however, only one of the preventive vaccine candidates tested in human efficacy trials afforded even modest protection (3–8). Therapeutic vaccine trials for HIV have also failed to show any sustainable impact on viral load and/or viral reservoirs (9–14). Vaccination strategies that generate sustained, high-quality, HIV-specific, adaptive immune responses may be a key component to future success (15–18).

Human trials of HIV vaccines that utilize vector-based antigen delivery approaches have primarily included Adenoviral and Pox vector platforms (3–11, 13). Additional viral and bacterial vectors, derived from cytomegalovirus (CMV), listeria monocytogenes, and HIV lentiviral (LV) constructs have shown potential in pre-clinical studies, but safety concerns have slowed their development (19–22).

In this review, we focus on recent advances in the design of HIV-derived LV vectors for vaccines to induce and augment adaptive immune responses. LV vectors have been explored extensively for gene therapy applications, including for treatment of HIV-1 infection (23, 24). By contrast, the field is considerably less developed regarding their use as HIV vaccines, though pre-clinical and early clinical data show promise (20, 21, 25–30). It is important to highlight certain distinctions between these two applications of LV vectors for HIV, which in large part reflect the genes delivered and desired target cell. LV vectors integrate and replicate in both dividing and non-dividing cells making them efficient vehicles to deliver therapeutic genes with long-lived expression. LV vector-based gene therapies against HIV aim to confer host resistance to infection through delivery of genetic information that interferes with HIV entry or replication and, therefore, primarily target hematopoietic stem cells (HPSCs) or T cells. By contrast, LV vaccines for HIV target antigen-presenting cells (APCs) to deliver HIV antigens that are efficiently presented on MHC molecules. Dendritic cells (DCs) are the ideal LV vaccine targets as they are the most potent APCs and uniquely able to initiate primary immune responses and stimulate robust and durable antigen-specific T cell responses (31). Moreover, the use of LV vectors as DC vaccines offers several advantages over other DC antigen-loading strategies, including more sustained antigen expression following integration into the target cell genomic DNA, endogenous production of antigen for more efficient MHC presentation, the ability to encode immunostimulatory genes and check point inhibitors to enhance T cell responses, and minimal vector immunity when using certain pseudotyped constructs [e.g., LV vectors pseudotyped with different vesicular stomatitis virus G envelope (VSV-G) serotypes] (20, 29).

Here, we review the pre-clinical and early clinical data regarding the use of LV vectors for DC vaccination strategies against HIV-1, and discuss ongoing research to overcome challenges relating to LV vector safety and efficacy. In particular, we focus on recent advances regarding improved LV vector targeting, transduction, and activation of DCs and other APCs to improve their immunostimulatory capacity and mitigate safety concerns.

## Overview of Pre-Clinical and Human Studies of LV Vectors for HIV Vaccines

Several pre-clinical studies have demonstrated that LV vectors are able to induce strong HIV-specific adaptive immune responses (20, 21, 25–29), and preliminary data from an early phase study in HIV-infected individuals support these findings (30). LV vectors encoding HIV-1 or SIV epitopes induced strong HIV-specific cytotoxic T lymphocytes (CTLs), as well as humoral responses in both mouse models and human *in vitro* studies (21, 25, 26, 28, 29). Notably, LV vectors have demonstrated superior immunogenicity when compared with other vaccine platforms in mice (27, 29, 32). An HIV-1-based LV vector encoding HIV Gag, Pol, and Rev (VRX1023) stimulated more potent and more durable mucosal and systemic cellular and humoral responses than administration of Ad5 HIV vectors (29). Additionally, no anti-vector immunity was detected against the HIV-1-based LV vector, allowing the use of a multiple injection approach to augment immune responses (29). In other disease models, LV vectors were similarly found superior in generating antigen-specific T cell responses in terms of both magnitude and longevity when compared to peptide-pulsed DC vaccines and peptide-adjuvant combinations (27, 32).

In non-human primate models of SIV, macaques that had been immunized with two doses of a VSV-G pseudotyped LV vector expressing SIVmac239 Gag and then challenged with rectal SIVmac251 were protected throughout the acute phase of infection (20). A single injection of the LV vector elicited strong and diverse Gag-specific T cell responses that peaked at 16 days post priming regardless of the dose used, whereas a second dose of non-cross-reactive VSV-G pseudotyped LV vectors administered 11 weeks later raised more robust and rapid Gag-specific T cell responses, albeit similar in breadth. Subsequent challenge of high-dose SIVmac251 resulted in infection in all animals; however, the acute phase of infection showed a reduction in viral replication by more than two orders of magnitude and protection against CD4 T cell (CD28+ CD95+) loss. Lower viral set points in vaccinated animals were also observed, though these were not statistically significant by day 49 post-infection (20).

A first-in-human, phase I/II randomized, controlled study in 38 HIV-infected individuals receiving antiretroviral therapy is currently underway (NCT02054286) (30). In this study, a non-replicative and self-inactivating LV vector encoding portions of HIV Gag, Pol, Nef proteins (TV01, Theravectys) is administered via two intramuscular (IM) injections spaced 8 weeks apart. Initial analysis revealed that TV01 is safe and highly immunogenic with CD4+ and CD8+ T cell responses to multiple vaccine-associated epitopes. These CD4+ and CD8+ T cell responses were found to be polyfunctional as evidenced by production of multiple cytokines and sustained for up to 24 weeks (30). Evaluation of ART interruption is reported to be underway, in addition to plans for a Phase II study.

Taken together, these pre-clinical and now early clinical findings support the potential therapeutic application of LV vectors as HIV vaccines. To improve upon the potency and safety of such vectors, it will be critical to use strategies that enhance the targeting, transduction efficiency, and stimulation of DCs and other APCs.

## DC-Targeting Approaches

**Figure 1:**
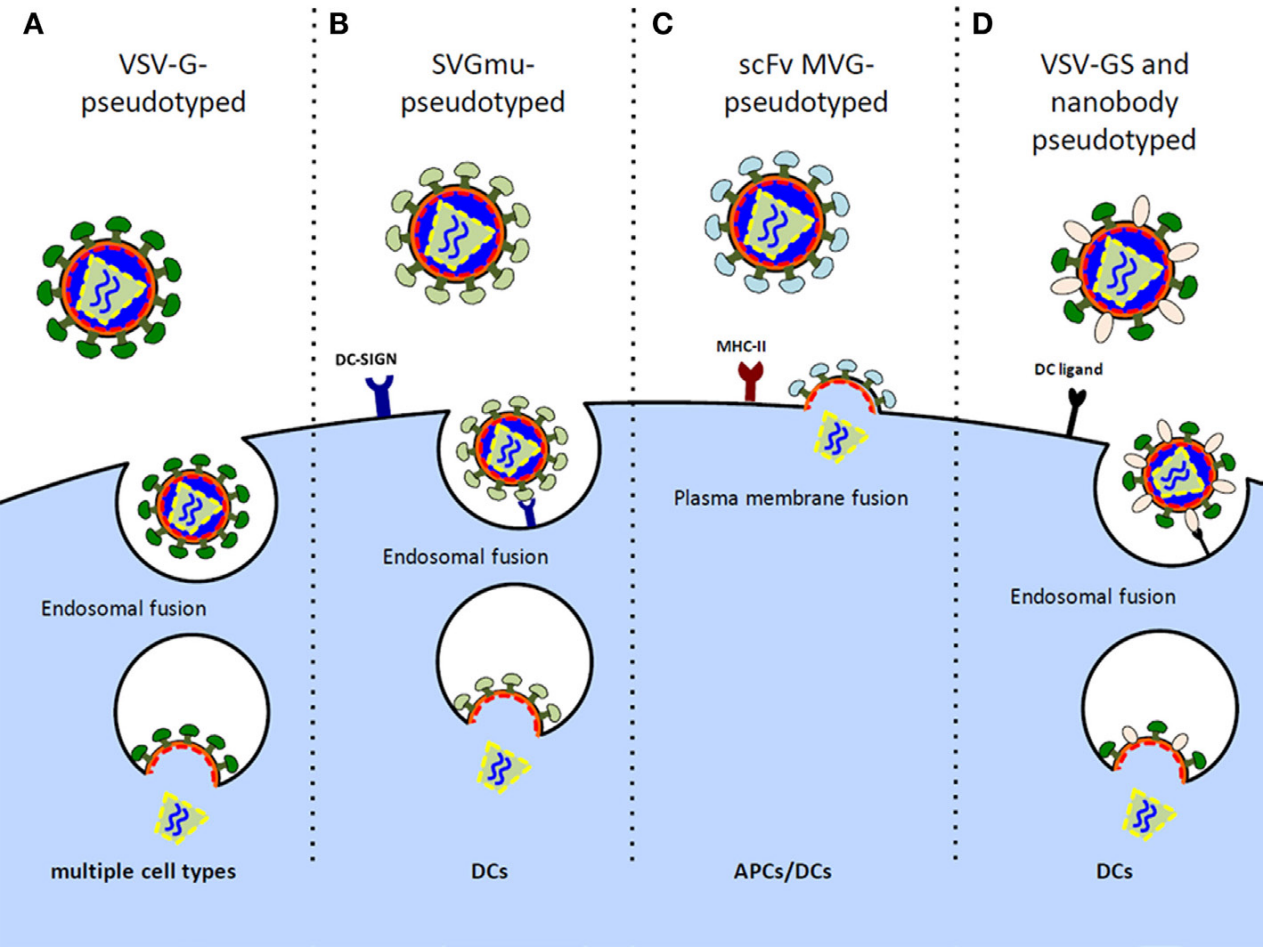
**Design of LV vectors to preferentially target DCs/APCs**. **(A)** Vesicular stomatitis virus G (VSV-G) glycoprotein-pseudotyped LV vectors possess diverse cell tropism, including transduction of non-dividing cells. **(B)** Sindbis virus glycoprotein (SVGmu)-pseudotyped LV vectors selectively target DCs by binding the endocytic receptor, DC-SIGN, while containing mutations in the heparan sulfate binding site to prevent transduction of other cell types. **(C)** LV vectors pseudotyped with measles virus glycoprotein (MVG) mediate direct cell entry via membrane fusion at the plasma membrane. These vectors can be engineered to display single-chain antibody (scFV) directed at MHC II to target transduction of APCs. **(D)** VSV-GS, a binding-defective, fusion competent VSV-G, and nanobody-pseudotyped LV virions are engineered to bind various DC ligands for targeted fusion.

Initial HIV-1-based LV vectors were generated as VSV-G glycoprotein pseudotypes to allow for production of highly infectious virus with a broad tropism for target cell transduction, including non-dividing cells (Figure 1A) (33). The use of other glycoproteins for pseudotyping has been explored with the intent of minimizing off-target effects, improving safety, and enhancing potency. LV vectors pseudotyped with a mutated Sindbis virus glycoprotein (SVGmu) confer a natural tropism toward human DCs as SVGmu selectively binds to the DC surface protein DC-SIGN (CD209) (Figure 1B) (34). Unlike standard laboratory-adapted Sindbis virus envelopes that target ubiquitously expressed heparan sulfate in addition to DC-SIGN, SVGmu contains mutations in the heparan sulfate binding site that prevent heparan sulfate-mediated cell entry. A single injection of an SVGmu-pseudotyped LV vector encoding tumor antigen or HIV-1 Gag in mice stimulated their DCs to induce a durable antitumor or HIV-1-specific immune response, respectively, while inducing only low levels of anti-vector neutralizing antibodies (34, 35). Moreover, prime/boost regimens consisting of either a heterologous DNA prime/SVGmu-LV-Gag boost or successive SVGmu-LV-Gag injections enhanced cellular and humoral responses and proved superior to a DNA prime/adenoviral vector boost immunization in terms of both the breadth and polyfunctionality of the vaccine-induced Gag-specific CD8+ and CD4+ T cells. Subsequent modifications of SVGmu, through amino acid substitutions in the receptor-binding site and restoration of the wild-type furin cleavage, improved proteolytic processing and virus maturation and led to enhanced DC-SIGN specificity and production yields (36).

Measles virus glycoproteins (MVGs), hemagglutinin (H) and fusion (F), have also been used to pseudotype LV vectors for DC targeting. A potential advantage over SVGmu and VSV-G-pseudotyped lentiviruses that require endocytosis for viral membrane fusion and cell entry (37) is that MVG-pseudotyped lentiviruses fuse at the plasma membrane for direct cell entry. The H glycoprotein of the measles virus is responsible for receptor recognition and confers a natural tropism for both the CD46 receptor expressed on all nucleated cells and the signaling lymphocyte activation molecule (SLAM) receptor constitutively expressed on memory T cells, thymocytes, select B cells, monocytes, and DCs (38). Remarkably, measles virus-pseudotyped vectors were shown to be fourfold more infectious in DCs compared to VSV-G-pseudotyped vectors at an MOI of 10 (39). Moreover, unlike VSV-G-pseudotyped lentiviruses that induced DC activation, pseudotyping with MVGs did not affect the maturation and activation status of the transduced DCs, thereby minimizing any unintended stimulation. In effort to develop APC-specific MVG-pseudotyped LV vectors, mutations were made in the H glycoprotein’s CD46 and SLAM recognition sites, and the glycoprotein was further engineered to display single-chain antibody (scFv) directed against major histocompatibility complex class II (MHC II) (Figure 1C) (40–42). Resultant LV vectors showed high *in vivo* DC specificity and induced significant CD4+ and CD8+ T cell responses, albeit, inferior to the VSV-G-pseudotyped vectors, likely owing to the impaired transduction efficiency and stability of the chimeric constructs. Mice immunized with a single injection of the HIV-1-derived LV vector pseudotyped with MHC II-targeted MVGs did, however, mount antigen-specific effector CD4+ and CD8+ T cells and establish T cell immune memory, suggesting their potential for clinical use (41).

An alternative approach to generating DC-specific LV vectors is to take advantage of the natural LV budding process and incorporate cell-targeting moieties at the viral surface. To this end, LV vectors were pseudotyped with a binding-defective, fusion-competent VSV-G glycoprotein (VSV-GS) and DC-specific single variable regions derived from camel IgG sequences, called nanobodies. The resultant virions selectively bind DC receptors to allow DC-specific membrane fusion (Figure 1D). This nanobody display technology has proven effective at targeting LV vectors to murine DCs both *in vitro* and *in situ* and has previously been well reviewed (43, 44).

A comparison of the different strategies used to develop LV vectors for DC-targeted delivery is summarized (Figure 1). These approaches carry inherent safety advantages over broadly tropic vectors, and it is likely that continued improvements in their stability, DC specificity, and transduction efficiency will advance their readiness for clinical testing.

## Strategies to Enhance DC Transduction

DC-targeting aside, the development of HIV-1-based LV vectors as DC vaccines has been limited by the low efficiency with which DCs are transduced. DCs express SAMHD1, a phosphohydrolase that depletes the cell of deoxynucleotide triphosphates, thereby halting infection of HIV-1-based vectors at the level of reverse transcription (45–47). HIV-2 and some SIV isolates encode the accessory protein Vpx that counteracts this block to infection by binding to SAMHD1 and recruiting an E3 ubiquitin ligase complex, CRL4, to mediate its proteasomal degradation (48, 49). However, HIV-1 does not encode Vpx and lacks a similar mechanism to counteract SAMHD1. Various strategies have, therefore, been attempted to deliver Vpx with HIV-1-based LV vectors in order to improve their ability to transduce DCs.

Initial studies co-administered LV virions with virus-like particles (VLPs) containing Vpx to facilitate SAMHD1 degradation and improve LV vector DC transduction efficiency (50). More recently, Sunseri et al. identified the 10 amino acid packaging motif in the P6 protein of SIVmac Gag that is required to package Vpx. Introduction of this motif into P6 of the HIV-1 Gag/Pol packaging vector allowed for the production of HIV-1 virions that contained a high copy number of Vpx molecules in its native location (51). The Vpx-packaged HIV-1 vectors infected DCs with a two-log increase in titer and allowed for the stable expression of transgenes or shRNA knock-down of target genes (52). In the absence of the Vpx-packaging motif, HIV-1-based LV vectors packaged only trace quantities of Vpx and the virus had much lower titers on DCs (21, 51, 52). An alternative approach to allow Vpx packaging was to fuse Vpx to a cSrc membrane-targeting domain (53). Co-transfection of the Vpx-cSrc expression plasmid during LV production allowed Vpx to be re-localized from the nucleus to the plasma membrane where it could be packaged into virion particles during virion assembly. Resultant Vpx-packaged lentiviruses encoding antigenic epitopes were shown to efficiently transduce DCs and stimulate expansion of antigen-specific CD8 T cells (54). It has been reported that Vpx-packaging is possible without any modification to the HIV-1 capsid or fusion to cSrc (36), though the Vpx-packaging efficiency is likely to be considerably improved by these systems and allow for more efficient DC transduction.

## Strategies to Enhance LV Vector Immunogenicity

In chronic infections, such as HIV, continuous antigen stimulation along with other factors leads to immunologic tolerance and CTL exhaustion. An advantage of using viral vectors for DC transduction is that the vectors can accommodate large gene inserts and express immunostimulatory proteins to serve as adjuvants for enhancing or regulating antigen-specific immune responses. Effective stimulation of antigen-specific T cells requires three signals: MHC-peptide complex recognition by antigen-specific T cell receptors (TCR), costimulation by DC surface molecules and their T cell ligands (i.e., DC CD80 and T cell CD28), and cytokine priming mediated by DC secretion of soluble cytokines. We previously showed that Vpx-packaged LV vectors expressing the DC stimulatory protein CD40 ligand (CD40L) fused to a single influenza or HIV viral epitope efficiently transduced DCs, causing the cells to mature and secrete proinflammatory and Th1 skewing cytokines (e.g., TNF-α, IL-12, and IL-6) that enhanced antigen presentation, and thus stimulated robust expansion of influenza or HIV-specific CTLs (21). Furthermore, the proinflammatory cytokines transiently released by DCs transduced with Vpx-vectors expressing CD40L reactivated latent HIV-1 provirus in latency models, supporting the use of such vectors in a two-pronged, “shock and kill” approach for reducing the latent reservoir and facilitating a functional cure (21). Goyvaerts et al. similarly capitalized on the ability of IL-12 to enhance expansion CD4+ Th1 cells and cytotoxic effector cells and showed that DCs transduced with LV vectors expressing IL-12-enhanced antigen-specific CTLs *in vivo* (55). Others developed LV vectors expressing antigenic epitopes in tandem with granulocyte-macrophage colony-stimulating factor (GM-CSF) and interleukin-4 (IL-4) or GM-CSF and IFN-α to enhance the longevity of transduced DCs and promote a more durable immune response (56–59).

In addition to enhancing immune responses, reversing immunologic tolerance by interfering with the programed death 1 (PD-1) pathway has been a well-studied means of restoring the function of exhausted antigen-specific CD8 T cells (60, 61). PD-1 is upregulated during chronic infection with HIV and its increased binding to its ligand, PD-1 ligand (PD-L1), mediates virus-specific CD8 T cell exhaustion by impairing the ability of T cells to become activated, proliferate, and produce cytokines. Blocking the PD-1/PD-1L inhibitory signal with an anti-PD-L1 antibody led to an enhanced HIV-1–specific CD8+ T cell response in mice immunized with DCs transduced with a LV vector expressing HIV-1 Gag (62). Interfering with PD-L1/PD-1 costimulation via LV vectors expressing soluble PD-1 or shRNA against PD-L1 also led to enhanced antigen-specific CTL responses (63–66), further highlighting the potential feasibility and importance of disrupting the PD-1/PD-L1 interaction in future LV vaccine strategies.

## Addressing Safety Concerns with LV Vaccine Vectors

Although DC targeting can minimize the off-target transduction of LV vectors, the use of HIV-derived LV vectors raises inherent safety concerns. These risks primarily relate to the formation of infectious virus and potential for host mutagenesis, and have been addressed in multiple ways to maximize safety. Several aspects regarding the design of LV vectors render the formation of replication-competent virus and subsequent infection exceedingly low (20). The risk of reconstituting pathogenic parental HIV-1 virus is eliminated by providing the only HIV-1 structural and enzymatic proteins required for the formation of LV virions in *trans* and using heterologous envelopes (e.g., VSV-G) such that LV vectors support only a single round infection. Furthermore, LV vector constructs generally lack accessory and regulatory genes, such as *vif, vpr, vpu*, and *nef*, that are required for efficient replication *in vivo* (67). Finally, self-inactivating vectors can also be designed that lack the 3′-LTR region of the viral promotor and enhancer.

The other major safety concern with LV vectors is the risk for potential mutagenesis caused by insertion of the viral genome into the host genome. The risk of insertional mutagenesis remains controversial but is likely very low as it has not been described with HIV-derived LV vectors in gene therapy trials to date (20, 23, 24). However, these concerns were highlighted in studies that used a retroviral vector derived from the Murine Moloney Leukemia Virus (MoMLV) in two SCID-X1 gene therapy trials (68, 69). In these studies, several cases of leukemia occurred post-treatment, thought to be caused by the transactivation of a proto-oncogene by a viral enhancer rather than a direct effect of integration (69). Options to offset this possibility include using a promotor that lacks enhancer activity, or the use of integration-deficient LV vectors (IDLVs) that cannot insert themselves into the host genome. It should also be highlighted that in the case of LV vectors for vaccination purposes as opposed the gene therapy, the likelihood of a detrimental effect caused by insertional mutagenesis is reduced as the transduced APCs do not proliferate and will likely be cleared by the subsequent CTLs that are formed.

The use of IDLVs generated by the expression of a catalytically inactive integrase is the most fail-safe means to mitigate any potential risk for insertional mutagenesis (70). Instead of inserting into the host genome, episomal DNA accumulates in the nucleus, resulting in transcription and efficient expression of the target gene (71). IDLV have been shown to be immunogenic with the ability to elicit both humoral and cell-mediated responses after IM and mucosal administration, although somewhat weaker than integrating LV vectors (72, 73). It remains poorly understood why IDLV have been less potent as vaccine vectors in pre-clinical studies compared with integrative LV vectors. Unlike integrated DNA, episomal DNA may be diluted in dividing cells over time. However, given that APCs/DCs are non-proliferating, decreased expression of target genes over time would seem less likely to impact their efficacy as vaccine vectors. Despite the weaker potency that has been observed when compared with integrative LV vectors, IDLV have been effective in stable transduction following IM administration in mice (74, 75) and has proven effective in multiple models of infection, including West Nile virus (76), HPV-associated tumors (77), and malaria (78). In terms of pathogens acquired via a mucosal route, including HIV, both mucosal immune responses and systemic immune responses are desirable for protection. Mucosal immunization using IDLV by various routes has shown promise in achieving protection at these surfaces in mouse models (79, 80). CD8+ T cell responses were observed in the gut in the lamina propria following IM vaccination with IDLV, but an adjuvanted sublingual protein boost was required in order to induce mucosal IgA responses (80). Intranasal (IN) vaccination with an IDLV expressing influenza nucleoprotein (NP) was compared to IM vaccination and both were found to induce NP-specific B and T cell responses, however, only the IN route protected from influenza virus infection (79).

A human vaccine trial combining these many safety measures in a phase I dose-escalation study in solid cancers is currently under way (NCT02122861) (81). This trial uses replication-incompetent, IDLV vectors expressing full-length NY-ESO-1, engineered to target DC-SIGN (LV305, Immune Design) and induce tumor-specific CTLs in patients with advanced NY-ESO-1 expressing melanoma, sarcoma, breast, lung, or ovarian cancers. Early results from dose escalation in 12 patients with sarcoma demonstrated safety up through the highest dose (10^10^ vg) administered intradermally (ID), with only grade I/II adverse events. The early immunogenicity from only the lowest dose group (10^8^ vg, *N* = 6) has been reported following receipt of 3–4 ID injections q3weeks, and found to generate strong T cell responses and some early evidence of antitumor effect (81).

## Conclusion

HIV-1-derived LV vectors are an effective tool for targeting DCs and inducing potent HIV-specific adaptive immune responses. The best means to preferentially target DCs to maximize both safety and immunogenicity of LV vectors is a matter of ongoing investigation and remains a key issue. A better understanding of HIV restriction factors, such as SAMHD1, and their counteraction by viral proteins, such as Vpx, has lead to the development of vectors with high DC transduction efficiencies and reinvigorated the DC vaccine field. Incorporation of immunostimulatory genes into LV vectors as adjuvants has further improved their immunogenicity. Additionally, the safety of LV vectors has been enhanced through developments in IDLVs and substantiated by their exploration in clinical trials. Taking together, therapeutic LV vector-based DC vaccines have the potential to alter the traditional vaccine landscape and may provide a means to contribute to functional cure strategies for HIV, as well as other diseases where antigenic targets have been identified.

## Author Contributions

EM and TN contributed equally to this mini-review in both research and writing.

## Conflict of Interest Statement

The authors declare that the research was conducted in the absence of any commercial or financial relationships that could be construed as a potential conflict of interest.
