# Corrigendum: Recent Advances in Lentiviral Vaccines for HIV-1 Infection

**DOI:** 10.3389/fimmu.2016.00354

**Published:** 2016-09-14

**Authors:** Thomas D. Norton, Elizabeth A. Miller

**Affiliations:** ^1^Department of Medicine, Division of Infectious Diseases, NYU School of Medicine, New York, NY, USA; ^2^Department of Medicine, Division of Infectious Diseases, Icahn School of Medicine at Mount Sinai, New York, NY, USA

**Keywords:** dendritic cells, lentiviral vectors, HIV-1, HIV-1 vaccines, SAMHD1, Vpx

In the original article, an error in the references in the section entitled “Addressing Safety Concerns with LV Vaccine Vectors” is present. Reference number 20 which is cited twice in this section is an error and should instead be Di Nunzio et al. (1).

The authors apologize for this. This error does not change the scientific conclusions of the article in any way.

## Conflict of Interest Statement

The authors declare that the research was conducted in the absence of any commercial or financial relationships that could be construed as a potential conflict of interest.
